# Stable tensor neural networks for efficient deep learning

**DOI:** 10.3389/fdata.2024.1363978

**Published:** 2024-05-30

**Authors:** Elizabeth Newman, Lior Horesh, Haim Avron, Misha E. Kilmer

**Affiliations:** ^1^Department of Mathematics, Emory University, Atlanta, GA, United States; ^2^Mathematics and Theoretical Computer Science, IBM TJ Watson Research Center, Yorktown, NY, United States; ^3^Department of Applied Mathematics, Tel Aviv University, Tel Aviv-Yafo, Israel; ^4^Department of Mathematics, Tufts University, Medford, MA, United States

**Keywords:** tensor algebra, deep learning, machine learning, image classification, inverse problems

## Abstract

Learning from complex, multidimensional data has become central to computational mathematics, and among the most successful high-dimensional function approximators are deep neural networks (DNNs). Training DNNs is posed as an optimization problem to learn network weights or parameters that well-approximate a mapping from input to target data. Multiway data or tensors arise naturally in myriad ways in deep learning, in particular as input data and as high-dimensional weights and features extracted by the network, with the latter often being a bottleneck in terms of speed and memory. In this work, we leverage tensor representations and processing to efficiently parameterize DNNs when learning from high-dimensional data. We propose tensor neural networks (t-NNs), a natural extension of traditional fully-connected networks, that can be trained efficiently in a reduced, yet more powerful parameter space. Our t-NNs are built upon matrix-mimetic tensor-tensor products, which retain algebraic properties of matrix multiplication while capturing high-dimensional correlations. Mimeticity enables t-NNs to inherit desirable properties of modern DNN architectures. We exemplify this by extending recent work on stable neural networks, which interpret DNNs as discretizations of differential equations, to our multidimensional framework. We provide empirical evidence of the parametric advantages of t-NNs on dimensionality reduction using autoencoders and classification using fully-connected and stable variants on benchmark imaging datasets MNIST and CIFAR-10.

## 1 Introduction

With the explosion of computing resources, including cloud-based storage and accessible advanced hardware, learning from large-scale, multiway data has become possible. Two distinct fields have emerged as the gold standards for handling multidimensional data: tensor analysis for featurization and compression and deep learning for high-dimensional function approximation. Both deep learning and tensor methods have achieved strong performance in image and video recognition (Vasilescu and Terzopoulos, 2002; Krizhevsky et al., 2012), medical imaging analysis (Omberg et al., 2007; Ronneberger et al., 2015), spatiotemporal weather analysis (Chattopadhyay et al., 2020; Li et al., 2020), and more. This work focuses on leveraging advantages of tensor methods to enhance deep learning design.

Fundamentally, deep learning approximates mappings from (high-dimensional) inputs (e.g., images) to targets (e.g., classes) using deep neural networks (DNNs), which are simply nonlinear, composite functions parameterized by learnable weights. Despite the success and flexibility of DNNs, the storage and computational costs to design and apply these models can be a significant impediment—there can be millions of network weights and learning requires an immense amount of time and top-of-the-line computational hardware (e.g., GPU clusters).

These computational challenges become bottlenecks for the classic feed-forward neural network, which builds DNNs using dense linear operators (matrices). Such operations uses network weights in an highly inefficient manner, and composing many of these dense matrices can require millions of weights, which is both computationally demanding and can lead to algorithmic problems, such as overfitting. To reduce these inefficiencies, we propose a new type of fully-connected layer that replaces dense linear operators with dense tensor operators. The proposed tensor operators can reduce the number of network weights by an order of magnitude, that leverage the inherent multidimensionality of the input data, and offer the potential for distributed computation. Thus, we call our architecture tensor neural networks (t-NNs).

The foundation of t-NNs is the ⋆_*M*_-product (pronounced “star-M”), a family of tensor-tensor products which induces an algebraic structure on a multidimensional space (Kernfeld et al., 2015). The ⋆_*M*_-framework provably encodes information more efficiently than traditional matrix algorithms (Kilmer et al., 2021) and has had success facial recognition (Hao et al., 2013), tomographic image reconstructions (Soltani et al., 2016; Newman and Kilmer, 2020), video completion (Zhang et al., 2014), image classification (Newman et al., 2018), and solving tensor linear systems (Ma and Molitor, 2022). We call the ⋆_*M*_-product *matrix-mimetic*; that is, familiar notions such as the identity and transpose are well-defined for the multilinear operation. The advantages of processing data multidimensionally including better leveraging inherit multiway structure and reducing the number of learnable network weights by an order of magnitude. The matrix-mimeticity enables the proposed t-NNs to naturally extend familiar deep learning concepts, such as backward propagation and loss functions, and non-trivial architectural designs to tensor space. We propose two additional extensions: tensor-based loss functions and a stable multidimensional framework, motivated by Haber and Ruthotto (2017), that brings topological advantages of featurization.

### 1.1 Our contributions

Because of the popularity of this area of research, we want to clarify the objectives and contributions of this paper from the outset. Our contributions are the following:

- Tensor algebra and processing for efficient parameterization: we introduce a basic framework for t-NNs, describe the associated tensor algebra, and demonstrate the inherit properties from stable network architectures. We also derive the training algorithm for t-NNs, leveraging matrix-mimeticity for elegant formulations. We show that this tensor parameterization, compared to an equivalent matrix approach, can reduce the number of weights by an order of magnitude.
- Tubal loss functions: our the algebraic structure imposed by the ⋆_*M*_-product is applied end-to-end. This includes defining new loss functions based on the outputs of the t-NN, which are no longer scalars, but the high-dimensional analog called *tubes*. This requires a new definition of *tubal functions*, and opens the door to a wide range of new evaluation metrics. These metrics offer more rigorous requirements to fit the training data, and hence can yield networks that generalize better.
- Stable t-NNs: we demonstrate how matrix-mimeticity preserves of desirable network architecture properties, specifically stability. This will enable the development of deeper, more expressive t-NNs.
- Open-source code: for transparency and to expand the use of t-NNs, we provide open-source at https://github.com/elizabethnewman/tnn.
- Scope: our goal is to explore a new algebraic structure imposed on neural networks and its the advantages over equivalent architectures. This paper serves as the introduction of t-NNs and, similar to the original neural networks, we consider fully-connected layers only. We acknowledge that to obtain state-of-the-art results, we would need tools like convolutional and subsampling layers and significant hyperparameter tuning; however, these are outside the scope of this paper. Convolutional layers apply multiple translation-invariant filters to extract local connections; our t-NNs examine the global structure of the data. Subsampling or pooling layers reduce the dimensionality of our data and hence provide multi-scale features; our t-NNs use no pooling in order to preserve the algebraic structure. We address extensions of t-NNs to convolutional and subsampling layers in the conclusions.

### 1.2 Organization

This paper is organized as follows. In Section 2, we give a brief outline of related work combining tensors and deep learning. In Section 3, we give the background notation on tensor-tensor products. In Section 4, we formally introduce tensor neural networks (t-NNs) and tubal loss functions. In Section 5, we extend t-NNs to stable architectures and outline a Hamiltonian-inspired architecture. In Section 6, we provide numerical support for using t-NNs over comparable traditional fully-connected neural networks. In Section 7, we discuss future work including implementations for higher-order data and new t-NN designs.

## 2 Related work

The high dimensional nature of neural network weights has driven the need to reduce the number of weights through structure. Early studies, such as LeCun et al. (1989), demonstrated that neural networks could learn faster from less data and generalize better by removing redundant weights. Following the observation, several works showed that structured weights, such as convolutions (Krizhevsky et al., 2012), low rank weight matrices (Denil et al., 2013), and Kronecker-structured matrices (Jagtap et al., 2022), could perform well with significantly fewer parameters.

Tensor methods for compression high dimensional data and operators grew in popularity concurrently with the development of structured operators for neural networks. Many popular tensor frameworks are designed to featurize multiway arrays (Tucker, 1966; Carroll and Chang, 1970; Harshman, 1970; de Lathauwer et al., 2000; Kolda and Bader, 2009) or to approximate a given high-dimensional operator (Oseledets, 2011; Cichocki et al., 2016). Because the weights and features of deep neural networks are notoriously high-dimensional, tensorized approaches have gained traction. In Novikov et al. (2015), the authors combine efficient tensor storage and processing schemes with DNN training, resulting up to seven times fewer network weights. This work specifically used the tensor train style of weight storage, which is notable for compression of very high dimensional data, but does not have linear algebraic motivations in this context. Further studies followed, such as Chien and Bao (2018) that used multiway operations to extract features convolutionally. This work computes a Tucker factorization of convolutional features rather than treating tensors as operators. Similar layer contraction approaches, called tensor regression layers, have appeared in works such as in Cao et al. (2017) and Kossaifi et al. (2020). These approaches utilize low-rank Tucker-based factorizations to successfully reduce the number of weights in a network without sacrificing performance. These are more similar in spirit to pooling layers of convolutional neural networks rather than operations that preserve multilinearity. Many more studies have connected tensors and neural networks, and we recommend the survey (Wang et al., 2023) for a more complete history of the intersection of the two fields.

As we eluded to in the previous paragraph, in this work, we take a notably different perspective on tensors. We consider tensors as *multiway operators* and process our layers under this tensor operation. This provides a linear algebraic structure that enables us to extend desirable neural network structure to high dimensions with ease. Because of our strong algebraic foundation, we are able to express forward and backward propagation simply; in comparison, other tensor frameworks require heavy indexing notation. We share and achieve the same goal as other tensor approaches of reducing the number of network weights.

## 3 Background and preliminaries

To motivate our multidimensional neural network design, we start by introducing our notation and the tensor algebra in which we work. We use Matlab indexing notation throughout the paper, such as selecting the *j*-th column of a matrix via **A**(:, *j*) or **A**_:, *j*_.

### 3.1 Tensor preliminaries

**Figure 1:**
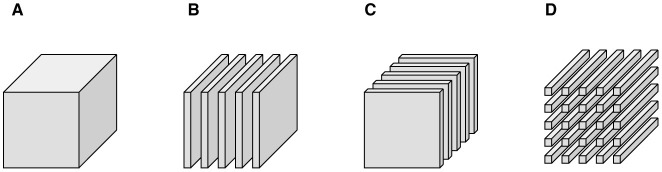
Tensor notation. **(A)** A is a third-order tensor, **(B)** ${\vec{A}}_{j}$ are lateral slices, **(C)** **A**^(*k*)^ are frontal slices, and **(D)** **a**_*ij*_ are tubes.

Let $A\in {\mathbb{R}}^{m_{1}\times m_{2}\times n}$ be a real-valued, third-order tensor. Fixing the third-dimension, *frontal slices* ${\text{A}}^{\left(k\right)}\in {\mathbb{R}}^{m_{1}\times m_{2}}$ are matrices for *k* = 1, …, *n*. Fixing the second-dimension, *lateral slices* ${\vec{A}}_{j}\in {\mathbb{R}}^{m_{1}\times 1\times n}$ are matrices oriented along the third dimension for *j* = 1, …, *m*_2_. Fixing the first and second dimensions, *tubes* ${\text{a}}_{ij}\in {\mathbb{R}}^{1\times 1\times n}$ are vectors oriented along the third dimension for *i* = 1, …, *m*_1_ and *j* = 1, …, *m*_2_. We depict these partitions in Figure 1. While this paper focuses on real-valued, third-order tensors (three indexes), we note all of the presented concepts generalize to higher-order and complex-valued tensors.

We interpret tensors as *t-linear operators* (Kilmer and Martin, 2011; Kernfeld et al., 2015). Through our operator lens, it is possible to define analogous matrix algebraic properties for tensors, such as orthogonality and rank. Thus, this framework has been described as *matrix-mimetic*. We describe the fundamental tools to understand how tensors operate for this paper, and refer the reader to Kilmer et al. (2013, 2021) and Kernfeld et al. (2015) for details about the underlying algebra.

We define a product to apply matrices along the third dimension of a tensor (i.e., along the tubes).

Definition 3.1 (mode-3 product). Given $A\in {\mathbb{R}}^{m_{1}\times m_{2}\times n}$ and **M** ∈ ℝ^ℓ × *n*^, the mode-3 product, denoted $\hat{A}\equiv A\times_{3}\text{M}$, outputs an *m*_1_×*m*_2_×ℓ tensor with entries


$$\begin{array}{l} \hat{A}\left(i_{1},i_{2},k\right)=\overset{n}{\underset{j=1}{\sum }}A\left(i_{1},i_{2},j\right)\text{M}\left(k,j\right) \end{array}$$

for *i*_1_ = 1, …, *m*_1_, *i*_2_ = 1, …, *m*_2_, and *k* = 1, …, ℓ.

The mode-3 product can be generalized along any mode; see Kolda and Bader (2009) for details.

Next, we define the facewise product to multiply the frontal slices of two third-order tensors in parallel.

Definition 3.2 (facewise product). Given $A\in {\mathbb{R}}^{m_{1}\times \ell \times n}$ and $B\in {\mathbb{R}}^{\ell \times m_{2}\times n}$, the facewise product, denoted C≡A▵B, returns an *m*_1_×*m*_2_×*n* tensor where


$$\begin{array}{l} {\text{C}}^{\left(k\right)}={\text{A}}^{\left(k\right)}{\text{B}}^{\left(k\right)} \end{array}$$

for *k* = 1, …, *n*.

Combining Definition 3.1 and Definition 3.2, we define our tensor operation, the ⋆_*M*_-product, as follows:

Definition 3.3 (⋆_*M*_-product). Given $A\in {\mathbb{R}}^{m_{1}\times \ell \times n}$, $B\in {\mathbb{R}}^{\ell \times m_{2}\times n}$, and an invertible *n*×*n* matrix **M**, the ⋆_*M*_-product outputs an *m*_1_×*m*_2_×*n* tensor of the following form:


$$\begin{array}{l} A{⋆}_{M}\;B=\left(\hat{A}▵\hat{B}\right)\times_{3}M^{-1}. \end{array}$$

where $\hat{X}\equiv X\times_{3}\text{M}$.

We say that A and B live in the spatial domain and $\hat{A}$ and $\hat{B}$ live in the transform domain. We perform the facewise product in the transform domain, then return to the spatial domain by applying **M**^−1^ along the tubes. If **M** is the identity matrix, the ⋆_*M*_-product is exactly facewise product. If **M** were the discrete Fourier transformation matrix (DFT), we obtain the t-product (Kilmer and Martin, 2011). In this case, the frontal slices of $\hat{A}$ correspond to different frequencies in the Fourier domain and are therefore decoupled.

We can interpret the ⋆_*M*_-product as a block-structured matrix product via


(1) $$\begin{array}{l} A{\;}_{⋆\text{M}}\;B\equiv \underset{\text{struct}(A)}{\underset{︸}{\left(M^{-1}⊗\;I_{m_{1}})\left[\begin{array}{cccc} {\hat{A}}^{\left(1\right)} & \; & \; & \;\\ \; & {\hat{A}}^{\left(2\right)} & \; & \;\\ \; & \; & \ddots & \;\\ \; & \; & \; & {\hat{A}}^{\left(n\right)} \end{array}\right](M⊗\;I_{\ell }\right)}}\underset{\text{unfold}(B)}{\underset{︸}{\left[\begin{array}{c} B^{\left(1\right)}\\ B^{\left(2\right)}\\ \vdots \\ B^{\left(n\right)} \end{array}\right]}} \end{array}$$

where ⊗ is the Kronecker product (Petersen and Pedersen, 2012). The block matrix structure, struct(A), depends on the choice of transformation, **M**. We consider two familiar examples: the facewise product (**M** = **I**_*n*_) and the t-product (**M** = **F**_*n*_, the DFT matrix):


(2a) $$\begin{array}{ll} M=I_{n} & \text{struct}(A)=\text{bdiag}(A)=\left[\begin{array}{cccc} A^{\left(1\right)} & \; & \; & \;\\ \; & A^{\left(2\right)} & \; & \;\\ \; & \; & \ddots & \;\\ \; & \; & \; & A^{\left(n\right)} \end{array}\right] \end{array}$$

(2b) $$\begin{array}{ll} \text{M}={\text{F}}_{n} & \text{struct}\left(A\right)=\text{bcirc}\left(A\right)=\left[\begin{array}{cccc} {\text{A}}^{\left(1\right)} & {\text{A}}^{\left(n\right)} & \cdots & {\text{A}}^{\left(2\right)}\\ {\text{A}}^{\left(2\right)} & {\text{A}}^{\left(1\right)} & \cdots & {\text{A}}^{\left(3\right)}\\ \vdots & \vdots & \ddots & \vdots \\ {\text{A}}^{\left(n\right)} & {\text{A}}^{\left(n-1\right)} & \cdots & {\text{A}}^{\left(1\right)} \end{array}\right]. \end{array}$$

While we never explicitly form Equation (2), the block structure will be helpful for subsequent analysis.

### 3.2 Matrix-mimetic tensor algebra

The ⋆_*M*_-product yields a well-defined algebraic structure. Specifically, suppose we have tubes **a**, **b**∈ℝ^1 × 1 × *n*^. Then,


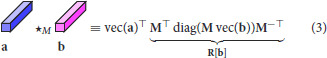


where $\text{vec}:{\mathbb{R}}^{1\times 1\times n}\to {\mathbb{R}}^{n\times 1}$ turns a tube into a column vector. The ⋆_*M*_-product of tubes (3) is equivalent to post-multiplying by the structured matrix, **R**[**b**]. Note that **R**[·] implicitly depends on the choice of **M**, but we omit explicitly writing this dependence for notational simplicity. The tubes form a matrix subalgebra which dictates the algebraic structure imposed on the high-dimensional space (Kernfeld et al., 2015). As a result, the ⋆_*M*_-product is *matrix-mimetic* and yields several familiar concepts.

Definition 3.4 (⋆_*M*_-identity tube). The identity tube **e** ∈ ℝ^1 × 1 × *n*^ under the ⋆_*M*_-product is


$$\begin{array}{l} \text{e}=1\times {\text{M}}^{-1}, \end{array}$$

where **1** is the 1 × 1 × *n* tube containing all ones.

This gives rise to the notion of an identity tensor.

Definition 3.5 (⋆_*M*_-identity tensor). A tensor $I\in {\mathbb{R}}^{m\times m\times n}$ is the identity tensor if I(i,i,:)=e for *i* = 1, …, *m* where **e** is the ⋆_*M*_-identity tube.

Note that if the size of third dimension is equal to one (i.e., *n* = 1), then Definition 3.5 collapses into the identity matrix. This is a hallmark of our ⋆_*M*_-framework and matrix-mimeticity; the product and definitions reduce to the equivalent matrix definitions when the third dimension is removed.

Definition 3.6 (⋆_*M*_-transpose). Given $A\in {\mathbb{R}}^{m_{1}\times m_{2}\times n}$, its transpose $B\equiv A^{⊤}\in {\mathbb{R}}^{m_{2}\times m_{1}\times n}$ is


$$\begin{array}{l} {\hat{\text{B}}}^{\left(k\right)}={\left({\hat{\text{A}}}^{\left(k\right)}\right)}^{H} \end{array}$$

for *k* = 1, …, *n*.

Note that if our transformation **M** is complex-valued, the transpose operator in the transform domain performs the conjugate transpose. However, because we are working with real-valued tensors in the spatial domain, the transpose will be real-valued as well.

## 4 Tensor neural networks (t-NNs)

In general, neural networks are parameterized mappings from an input space Y to the target space C. These mappings are composite functions of the form


(4) $$F_{\text{NN}}(\cdot ,\theta )\equiv f_{d}(\cdots f_{2}(f_{1}(\cdot ,\theta_{1}),\theta_{2})\cdots ),\;\theta_{d}).$$

Each subfunction *f*_*j*_(·, ***θ***_*j*_) for *j* = 1, …, *d* is called a *layer*. The goal is to find a good set of weights ***θ*** ≡ (***θ***_1_, …, ***θ***_*d*_) ∈ Θ such that *F*_NN_(**y**, ***θ***) ≈ *c* for all input-target pairs (y,c)⊂D. Here, Θ is the parameter space and D⊂Y×C is the data space.

The most common layer of feed forward neural networks (4) consists of an affine transformation and pointwise nonlinearity of the form


(5) $$\begin{array}{l} {\text{y}}_{j}=f_{j}\left({\text{y}}_{j-1},{\text{θ}}_{j}\right)=\sigma_{j}\left({\text{W}}_{j}{\text{y}}_{j-1}+{\text{b}}_{j}\right) \end{array}$$

where ${\text{W}}_{j}\in {\mathbb{R}}^{m_{j}\times m_{j-1}}$ is a weight matrix, ${\text{b}}_{j}\in {\mathbb{R}}^{m_{j}}$ is a bias vector, and $\sigma_{j}:\mathbb{R}\to \mathbb{R}$ is a one-dimensional nonlinear activation function, applied entrywise. In practice, activation functions are monotonic, such as the sigmoid function, σ(*x*) = 1/(1+*e*^−*x*^), or Rectified Linear Unit (ReLU), σ(*x*) = max(*x*, 0). We call **y**_*j*_ the *features* of layer *j* and ${\text{y}}_{0}\in Y$ are the input features. Notationally, we use ***θ***_*j*_ ≡ (**W**_*j*_, **b**_*j*_) to collect all of the learnable weights for layer *j*.

### 4.1 Improved parameterization with the ⋆_*M*_-product

When designing a neural network, we seek to balance a simple parameter space with an expressive feature space. However, traditionally fully-connected layers like (5) use parameters in a highly inefficient manner. We propose new tensor fully-connected layers for a more efficient parametrization while still creating a rich feature space. Specifically, we consider the following tensor forward propagation scheme:


(6) $$\begin{array}{l} {\vec{Y}}_{j}=\sigma_{j}\left(W_{j}{⋆}_{M}{\vec{Y}}_{j-1}+{\vec{B}}_{j}\right), \end{array}$$

for *j* = 1, …, *d*. Suppose our input features ${\vec{Y}}_{0}=\vec{Y}\in Y$ is of size *m*_0_×1 × *n*. Here, the weight tensor $W_{j}$ is of size *m*_*j*+1_×*m*_*j*_×*n* and the bias ${\vec{B}}_{j}$ is of size *m*_*j*+1_×1 × *n*. The forward propagation through Equation (6) results in a tensor neural network *F*_tnn_(·, ***θ***) where $\text{θ}\equiv {\left(W_{j},{\vec{B}}_{j}\right)}_{j=1}^{d}$.

**Figure 2:**
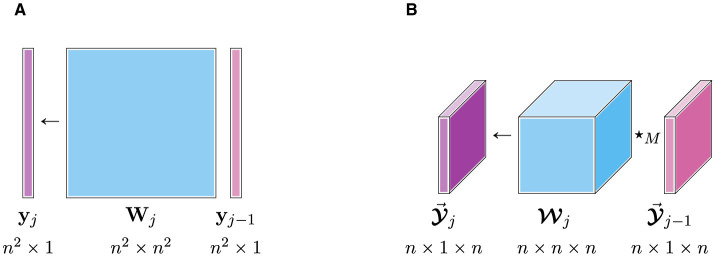
Comparison of network parameterizations to preserve the number of features of layer *j*−1 and layer *j*. The matrix mapping requires *n*^4^ weights in **W**_*j*_ and tensor mapping requires *n*^3^ weights in $W_{j}$. **(A)** Matrix linear mapping. **(B)** Tensor ⋆_*M*_-mapping.

Through the illustration in Figure 2, we depict the number of weight parameters required to preserve the size of our feature space using either a dense matrix (Figure 2A) or a dense tensor under the ⋆_*M*_-product (Figure 2B). Using the ⋆_*M*_-algebra, we can reduce the number of weight parameters by a factor of *n* while maintaining the same number of features (i.e., maintaining a rich feature space). Beyond the parametric advantages, the multilinear ⋆_*M*_-product incorporates the structure of the data into the features. This enables our t-NNs to extract more meaningful features, and hence improve the richness of our feature space.

### 4.2 The training problem

Training a (tensor) neural network is posed as a stochastic optimization problem given by


(7) $$\begin{array}{l} \underset{\text{θ}\in \Theta }{\min}\;𝔼L\left(F_{\text{tnn}}\left(\vec{Y},\text{θ}\right),c\right)+\lambda R\left(\text{θ}\right), \end{array}$$

where $L:{\mathbb{R}}^{m_{d+1}\times 1\times n}\times C\to \mathbb{R}$ is the loss function that measures the misfit between the network prediction and the true target. The expectation is taken over all input-target pairs $\left(\vec{Y},c\right)\in D$. The additional function R:Θ→ℝ regularizes the weights to promote desirable properties (e.g., smoothness), weighted by a regularization parameter λ > 0.

### 4.3 Tubal loss (t-loss) functions

The loss function is chosen based on the given task. For regression, we often use mean squared error, and for classification, which is the focus of this paper, we often use cross entropy. Cross entropy loss, related to the Kullback–Leibler (KL) divergence (Kullback and Leibler, 1951), measures the distance between two probability distributions. In practice, we first transform the network outputs into a set of probabilities using exponential normalization. Specifically, we use the softmax function $h:{\mathbb{R}}^{p}\to \Delta^{p}$, defined entrywise as


(8) $$\begin{array}{l} {\left[h\left(\text{x}\right)\right]}_{i}=\frac{e^{x_{i}}}{\overset{p}{\underset{j=1}{\sum }}e^{x_{j}}}\;\text{for}i=1,\ldots ,p, \end{array}$$

where Δ^*p*^ is the *p*-dimensional unit simplex.

To preserve the algebraic integrity of t-NNs, we introduce a tubal variant of the softmax function. Drawing inspiration from Lund (2020), a tubal function, we start by defining tubal functions generally.

Definition 4.1 (tubal function). Given **b** ∈ ℝ^1 × 1 × *n*^, a *tubal function* $f:{\mathbb{R}}^{1\times 1\times n}\to {\mathbb{R}}^{1\times 1\times n}$ acts on the action of **b** under the ⋆_*M*_-product; that is,


(9) $$\begin{array}{l} \underset{\text{tubal function}}{f\left(\text{b}\right)}\equiv \underset{\text{matrix function}}{f\left(\text{R}\left[\text{b}\right]\right)}\equiv {\text{M}}^{⊤}\underset{\text{pointwise function}}{f\left(\text{M}\text{vec}\left(\text{b}\right)\right)}{\text{M}}^{-⊤} \end{array}$$

In practice, a tubal function is applied pointwise in the transform domain.

We note that the pointwise function in Definition 4.1 is equivalent to applying a matrix function to the eigenvalues of the matrix **R**[**b**]. We provide a visualization of the effects of tubal functions compared to applying an entry-wise function in Example 4.2.

**Figure 3:**
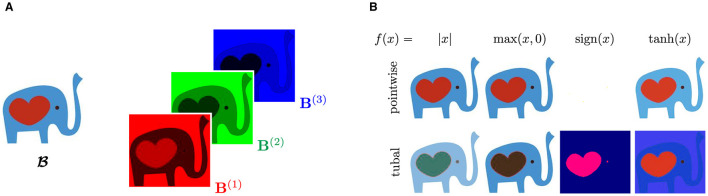
Comparison of entry-wise and tubal functions applied to roughly piecewise constant image. **(A)** Image and color channels of elephant image. The elephant is almost piecewise constant with easily distinguishable RGB values. **(B)** Effects of tubal functions under the transformation (10). Images are rescaled between 0 and 1 after applying the respective function.

Example 4.2 (Visualizations of tubal functions). Consider the following RGB image $B\in {\mathbb{R}}^{150\times 169\times 3}$ of a Tufts Community Apeal elephant (TCA, 2023), where 3 is the number of color channels (Figure 3A). We rescale each entry of B between 0 to 1 and consider the following transformation matrix:


(10) $$\begin{array}{l} M=\left(\begin{array}{ccc} -1 & 1 & 1\\ 0 & 1 & 1\\ 0 & 0 & 1 \end{array}\right)\;\text{and}\;M^{-1}=\left(\begin{array}{ccc} -1 & 1 & 0\\ 0 & 1 & -1\\ 0 & 0 & 1 \end{array}\right). \end{array}$$

To illustrate the effect of using tubal functions, we compare applying various functions as pointwise functions (i.e., independent of **M**) and as tubal functions using Equation (9) in Figure 3B.

Tubal functions are able to capture shared patterns among the tubes that entrywise operators ignore. Each choice of tubal function highlights different features of the image, such as the body with *f*(*x*) = max(*x*, 0) and the heart with *f*(*x*) = tanh(*x*). In comparison, the entrywise counterparts do not bring new insights or structure. A striking difference occurs for *f*(*x*) = sign(*x*). The entrywise operator turns all pixels white because the RGB entries are all nonnegative. In contrast, the tubal equivalent is applied in the transform domain, and hence reveals meaningful features of the image.

Note that the results depend on the chosen tubal function and transform **M**. We deliberately chose **M** to emphasize green and blue channels and diminish the effect of the red channel, enabling easy-to-interpret distinctions between tubal and entrywise functions.

We can now define a tubal softmax function based on the traditional softmax function in (8).

Definition 4.3 (tubal softmax). Consider a lateral slice $\vec{X}\in {\mathbb{R}}^{p\times 1\times n}$ as a *p*×1 vector of 1 × 1 × *n* tubes. Then, the tubal softmax function $h:{\mathbb{R}}^{p\times 1\times n}\to \Delta^{p\times 1\times n}$ performs the following mapping:


$$\begin{array}{l} {\left[h\left(\vec{X}\right)\right]}_{i}={\left(\overset{p}{\underset{j=1}{\sum }}\exp\left({\text{x}}_{j}\right)\right)}^{-1}{⋆}_{M}\exp\left({\text{x}}_{i}\right) \end{array}$$

for *i* = 1, …, *p* where $h\left(\vec{X}\right)\in {\mathbb{R}}^{p\times 1\times n}$ and the exponential functions are applied as tubal functions. Here, Δ^*p*×1 × *n*^ is the tubal-equivalent of *p*-dimensional unit simplex.

We interpret $h\left(\vec{X}\right)$ as a vector of “tubal probabilities” in that the tubes sum to the identity tube


$$\begin{array}{l} \overset{p}{\underset{i=1}{\sum }}{\left[h\left(\vec{X}\right)\right]}_{i}=\overset{p}{\underset{i=1}{\sum }}\left[{\left(\overset{p}{\underset{j=1}{\sum }}\exp\left({\text{x}}_{j}\right)\right)}^{-1}{⋆}_{M}\exp\left({\text{x}}_{i}\right)\right]\\ \;={\left(\overset{p}{\underset{j=1}{\sum }}\exp\left({\text{x}}_{j}\right)\right)}^{-1}{⋆}_{M}\left[\overset{p}{\underset{i=1}{\sum }}\exp\left({\text{x}}_{i}\right)\right]\\ \;=\text{e}. \end{array}$$

Through Definition 4.3, we demonstrate the parallels between tubal functions and traditional functions; the similarities are a direct consequence of a matrix-mimetic tensor framework.

The last step to define the tubal cross entropy function that converts the output of tubal function to a scalar. Recall, traditional cross entropy $L_{\text{ce}}:{\mathbb{R}}^{p}\times \left\{1,\ldots ,p\right\}\to {\mathbb{R}}_{+}$ for one sample is given by


(11) $$\begin{array}{l} L_{\text{ce}}\left(\text{x},c\right)=-\log{\left[h\left(\text{x}\right)\right]}_{c} \end{array}$$

where **x** is the output of the network, *c* is the corresponding target class, and *h* is the softmax function. We generalize (11) to a tubal variant as follows:

Definition 4.4 (tubal cross entropy (t-cross entropy)). The *tubal cross entropy function* $L_{\text{tce}}:\Delta^{p\times 1\times n}\times \left\{1,\ldots ,p\right\}\to {\mathbb{R}}_{+}$ is given by


$$\begin{array}{l} L_{\text{tce}}\left(\vec{X},c\right)=-||\text{vec}\left(\log{\left[h\left(\vec{X}\right)\right]}_{c,1,:}\right){||}_{q} \end{array}$$

where *h* is the tubal softmax function, log is applied as a tubal function, *c* is the index corresponding to the target class, and ||·||_*q*_ is a vector norm.

The intuition behind Definition 4.4 is the following. If we have good features $\vec{X}$, then ${\left[h\left(\vec{X}\right)\right]}_{c,1,:}\approx \text{e}$, the identity tube, and the remaining tubes will be closer to 0. In the transform domain, ${\left[h\left(\vec{X}\right)\times \text{M}\right]}_{c,1,:}\approx 1$ and the remaining entries will be close to zero. When we apply the log pointwise in the transform domain, the tube $\log{\left[h\left(\vec{X}\right)\times \text{M}\right]}_{c,1,:}\approx 0$ and the remaining entries will be large negative numbers. As a result, *L*_tce_ is smallest when the ${\left[h\left(\vec{X}\right)\right]}_{c,1,:}\approx \text{e}$, as desired.

In practice, if *q*-norm corresponds to a finite integer, we can instead use $||\log{\left[h\left(\vec{X}\right)\right]}_{c,1,:}|{|}_{q}^{q}$ for t-cross entropy for easier derivative computations. For numerical benefits when training, we consider normalized versions based on the number of tubal entries, e.g., multiply by 1/*n*. These suggested modifications should not change performance in theory, but could change preferred training hyperparameters.

### 4.4 Backward propagation with t-NNs

The workhorse of neural network training is backward propagation (Rumelhart et al., 1986; Bengio et al., 1994; Shalev-Shwartz et al., 2017; Nielsen, 2018), a method to calculate the gradient of the objective function (7) with respect to the weights. With gradient information, one can apply standard stochastic gradient optimization techniques to train.

In the ⋆_*M*_-framework, for an orthogonal transformation **M**, the backpropagation formulas are analogous to the matrix case. For example, the derivatives of the ⋆_*M*_-product are


(12) $$\begin{array}{r} \frac{\partial }{\partial \vec{Y}}\left[W{⋆}_{M}\vec{Y}\right]=W^{⊤}{⋆}_{M}\partial \vec{Y}\\ \text{and}\;\;\frac{\partial }{\partial W}\left[W{⋆}_{M}\vec{Y}\right]=\partial W{⋆}_{M}{\vec{Y}}^{⊤} \end{array}$$

where ∂X indicates a direction or perturbation of the same size as X. For full details on the derivation, we refer the reader to Newman (2019).

The simplicity of the back-propagation formulas (12) is one of the hallmarks of our choice of leveraging a matrix-mimetic tensor framework. Other tensor-based neural network designs (Wang et al., 2023) often require complicated indexing and non-traditional notation which, in addition to being cumbersome, can preclude extending more sophisticated neural network architectures to higher dimensions. The ⋆_*M*_-framework yields derivative formulations that are easy to interpret, implement, and analyze.

## 5 Stable t-NNs

As the depth (number of layers) of a network increases, gradient-based training is subject to numerical instability such as vanishing or exploding gradient problem (Bengio et al., 1994; Shalev-Shwartz et al., 2017). To avoid these instabilities, one can interpret deep neural networks as discretizations of differential equations (Ee, 2017; Haber and Ruthotto, 2017; Haber et al., 2018) and analyze the stability of forward propagation as well as the well-posedness of the learning problem; i.e., whether the classifying function depend continuously on the initialization of the parameters (Ascher, 2010). By ensuring stability and well-posedness, networks can generalize better to similar data and can classify data more robustly.

We emphasize that the notion of stability is related to the formal numerical analysis definition in the Lyapunov sense (i.e., stability of dynamical systems). This is a property of the model itself and independent of the data. From a statistical and foundational learning theory perspective, neural networks are typically over-parameterized models, which tend to overfit. In this context, stability can promote better generalization by imposing constraints of the structure of the weight matrices, effectively reducing the number of degrees of freedom. The tensorial structure imposes additional constraints and further reduces the number of parameters, which can again lead to better generalization.

### 5.1 Well-posed learning problem criterion

Consider the residual neural network (He et al., 2016) with tensor operations, given by


(13) $$\begin{array}{l} {\vec{Y}}_{j}={\vec{Y}}_{j-1}+h\sigma \left(W_{j}{⋆}_{M}{\vec{Y}}_{j-1}+{\vec{B}}_{j}\right)\;\text{for}j=1,\ldots ,d, \end{array}$$

where ${\vec{Y}}_{j}\in {\mathbb{R}}^{m\times 1\times n}$, $W_{j}\in {\mathbb{R}}^{m\times m\times n}$, and ${\vec{B}}_{j}\in {\mathbb{R}}^{m\times 1\times n}$. With the addition of the step size *h*, we can interpret Equation (13) as a forward Euler discretization of the continuous ordinary differential equation (ODE)


(14) $$\begin{array}{l} \frac{d\vec{Y}\left(t\right)}{dt}=\sigma \left(W\left(t\right){⋆}_{M}\vec{Y}\left(t\right)+\vec{B}\left(t\right)\right)\;\text{with }\vec{Y}\left(0\right)={\vec{Y}}_{0} \end{array}$$

for all *t* ∈ [0, *T*] where *T* is the final time corresponding to the depth of the discretized network.

The stability of non-autonomous ODEs like (14) depends on the eigenvalues of the Jacobian with repsect to the features. To perform analogous analysis for tensor operators, it is useful to consider the equivalent block matrix version of the ⋆_*M*_-product in Equation (1) where $W⋆M\vec{Y}\equiv \text{struct}\left(W\right)\text{unfold}\left(\vec{Y}\right)$. It follows that we can matricize (14) via


(15) $$\begin{array}{l} \frac{d}{dt}\text{unfold}\left(\vec{Y}\left(t\right)\right)=\sigma \left(\text{struct}\left(W\left(t\right)\right)\text{unfold}\left(\vec{Y}\left(t\right)\right)+\text{unfold}\left(\vec{B}\left(t\right)\right)\right). \end{array}$$

The Jacobian of the matricized system (15) with respect to $\text{unfold}\left(\vec{Y}\left(t\right)\right)$ is


$$\begin{array}{l} \text{J}\left(t\right)=\text{diag}\left(\sigma^{\prime }\left(\text{x}\left(t\right)\right)\right)\text{struct}\left(W\left(t\right)\right) \end{array}$$

where $\text{x}\left(t\right)=\text{struct}\left(W\left(t\right)\right)\text{unfold}\left(\vec{Y}\left(t\right)\right)+\text{unfold}\left(\vec{B}\left(t\right)\right)$ and **J**(*t*) ∈ ℝ^*mn*×*mn*^. In most cases, the activation function σ is non-decreasing, and thus the entries in diag(σ′(**x**(*t*))) are nonnegative. As a result, the stability and well-posedness of the ODE relies on the eigenvalues of struct(W(t)). As described in Haber and Ruthotto (2017), the learning problem is well-posed if


(16) $$\begin{array}{l} \text{Re}\left(\lambda_{i}\left(\text{struct}\left(W\left(t\right)\right)\right)\right)\approx 0\;\text{for}i=1,\ldots ,mn, \end{array}$$

where λ_*i*_(**A**) is the *i*-th eigenvalue of **A**. This criterion implies that the imaginary part of the eigenvalues drive the dynamics, promoting rotational movement of features. This produces stable forward propagation that avoids features diverging and prevents inputs from distinct classes from converging to indistinguishable points; the latter would lead to ill-posed back propagation and hence an ill-posed learning problem.

We can be more concrete about the eigenvalues because in Equation (1), struct(W(t)) is block-diagonalized in the transform domain. Thus, we can equivalently write Equation (16) as follows:


(17) $$\begin{array}{l} \text{Re}\left(\lambda_{i}\left({\hat{\text{W}}}^{\left(k\right)}\left(t\right)\right)\right)\approx 0 \end{array}$$

for *i* = 1, …, *m* and *k* = 1, …, *n*. In short, if the eigenvalues of each frontal slice in the transform domain have a real part close to zero, the t-NN learning problem will be well-posed, save one more requirement.

A subtle, yet important requirement for stability is that the weights change gradually over time (i.e., layers). This ensures that small perturbations of the weights yield small perturbations of the features. To promote this desired behavior, we imposed a smoothing regularizer in (discrete) time via


(18) $$\begin{array}{l} R_{\text{smooth}}\left({\left\{W_{j},{\vec{B}}_{j}\right\}}_{j=1}^{d}\right)=\overset{d-1}{\underset{j=1}{\sum }}||W_{j+1}-W_{j}{||}_{F}^{2}+||{\vec{B}}_{j+1}-{\vec{B}}_{j}{||}_{F}^{2}. \end{array}$$

### 5.2 Hamiltonian-inspired stable t-NNs

While theoretically useful, it is impractical to evaluate the eigenvalues of the weight tensors to satisfy the well-posedness condition (17) as we train a network. Instead, motivated by the presentation in Haber and Ruthotto (2017), we implement a forward propagation that inherently satisfies (17) independent of the weights. This forward propagation scheme is inspired by Hamiltonian dynamics, which we briefly describe and refer to Ascher (2010) and Brooks et al. (2011) for further details. We define a Hamiltonian as follows:

Definition 5.1 (Hamiltonian). Let $\text{y}\left(t\right)\in {\mathbb{R}}^{m_{y}\times 1}$, $\text{z}\left(t\right)\in {\mathbb{R}}^{m_{z}\times 1}$, and *t* ∈ [0, *T*]. A Hamiltonian $H:{\mathbb{R}}^{m_{y}\times 1}\times {\mathbb{R}}^{m_{z}\times 1}\times \left[0,T\right]\to \mathbb{R}$ is a system governed by the following dynamics:


$$\begin{array}{l} \frac{d\text{y}}{dt}=\nabla_{\text{z}}H\left(\text{y}\left(t\right),\text{z}\left(t\right),t\right)\;\text{and }\frac{d\text{z}}{dt}=-\nabla_{\text{y}}H\left(\text{y}\left(t\right),\text{z}\left(t\right),t\right). \end{array}$$

Intuitively, the Hamiltonian *H* describes the total energy of the system with **y** as the position and **z** as the momentum or velocity. We can separate total energy into potential energy *U* and kinetic energy *T*; that is, *H*(**y**, **z**, *t*) = *U*(**y**)+*T*(**z**). This separability ensures the Hamiltonian dynamics conserve energy; i.e.,


(19) $$\begin{array}{l} \frac{dH}{dt}=\frac{d\text{y}}{dt}\nabla_{\text{y}}H+\frac{d\text{z}}{dt}\nabla_{\text{z}}H=0 \end{array}$$

In terms of neural networks, energy conservation (19) ensures that network features are preserved during forward propagation, thereby avoiding the issue of exploding/vanishing gradients and enabling the use of deeper networks. Additionally, Hamiltonians are symplectic or volume-preserving in the sense that the dynamics are divergence-free. For neural networks, this ensures the distance between features does not change significantly, avoiding converging and diverging behaviors. We also note that Hamiltonians are time-reversible. This ensures that if we have well-posed dynamics during forward propagation, we will have similar dynamics for backward propagation.

### 5.3 Discretizing Hamiltonians with leapfrog integration

To preserve the benefits of Hamiltonians in the discretized setting, we symmetrize the Hamiltonian (Definition 5.1) and use a leapfrog integration method (Skeel, 1993; Ascher, 2010; Haber and Ruthotto, 2017). For t-NNs, we write the new system in terms of the ⋆_*M*_-product (with slight abuse of notation)


(20) $$\begin{array}{l} \frac{d}{dt}\left[\begin{array}{c} \vec{Y}\left(t\right)\\ \vec{Z}\left(t\right) \end{array}\right]=\sigma \left(\left[\begin{array}{cc} 0 & W\left(t\right)\\ -W{\left(t\right)}^{⊤} & 0 \end{array}\right]{⋆}_{M}\left[\begin{array}{c} \vec{Y}\left(t\right)\\ \vec{Z}\left(t\right) \end{array}\right]+\text{b}\left(t\right)\right) \end{array}$$

where $\vec{Y}\left(0\right)={\vec{Y}}_{0}$ and $\vec{Z}\left(0\right)=0$. Here, $\vec{Y}\left(t\right)$ indicates the data features and $\vec{Z}\left(t\right)$ is an auxilary variable not related to the data directly. We add the bias tube, **b**(*t*), to each element. The equivalent matricized version of Equation (20) is


(21) $$\begin{array}{l} \frac{d}{dt}\left[\begin{array}{c} \text{unfold}(\vec{Y}(t))\\ \text{unfold}(\vec{Z}(t)) \end{array}\right]=\sigma (\left[\begin{array}{cc} 0 & \text{struct}(W(t))\\ -\text{struct}{\left(W(t)\right)}^{⊤} & 0 \end{array}\right]\\ \;\left[\begin{array}{c} \text{unfold}(\vec{Y}(t))\\ \text{unfold}(\vec{Z}(t)) \end{array}\right]+\;\text{b}(t)). \end{array}$$

The (matricized) system (21) is inherently stable, independent of the weight tensors W(t), because of the block antisymmetric structure. The eigenvalues of antisymmetric matrices are purely imaginary, which exactly satisfy the stability condition in Equation (17).

We discretize Equation (20) using the leapfrog method, a symplectic integration technique, defined as


(22a) $$\begin{array}{l} {\vec{Z}}_{j+\frac{1}{2}}={\vec{Z}}_{j-\frac{1}{2}}-h\sigma \left(W_{j+1}^{⊤}{⋆}_{M}{\vec{Y}}_{j}+{\text{b}}_{j+1}\right) \end{array}$$

(22b) $$\begin{array}{l} {\vec{Y}}_{j+1}={\vec{Y}}_{j}+h\sigma \left(W_{j+1}{⋆}_{M}{\vec{Z}}_{j+\frac{1}{2}}+{\text{b}}_{j+1}\right) \end{array}$$

for *j* = 0, …, *d*−1. We demonstrate the benefits of stable forward propagation (22) in Example 5.2.

Example 5.2 (Trajectories of stable t-NNs). We construct a dataset in ℝ^3^ randomly drawn from a multivariate normal distribution with mean of 0 and a covariance matrix of 3**I**_3_. The points are divided into three classes based on distance to the origin with yellow points inside a sphere with radius *r* = 3.5, green points inside a sphere with radius *R* = 5.5, and purple points outside both spheres. We train with 1200 data points and store the data as 1 × 1 × 3 tubes.

We forward propagate using one of two integrators with weights and biases as 1 × 1 × 3 tubes:


(23a) $$\begin{array}{ll} \text{Forward Euler} & {\text{y}}_{j+1}={\text{y}}_{j}+h\sigma \left({\text{w}}_{j+1}{⋆}_{M}{\text{y}}_{j}+{\text{b}}_{j+1}\right) \end{array}$$

(23b) $$\text{Leapfrog }\left\{ \begin{array}{l} z_{j+\frac{1}{2}}=z_{j-\frac{1}{2}}-h\sigma (w_{j+1}^{⊤}{⋆}_{M}y_{j}+b_{j+1})\\ y_{j+1}=y_{j}+h\sigma (w_{j+1}{⋆}_{M}z_{j+\frac{1}{2}}+b_{j+1}) \end{array}\right.$$

**Figure 4:**
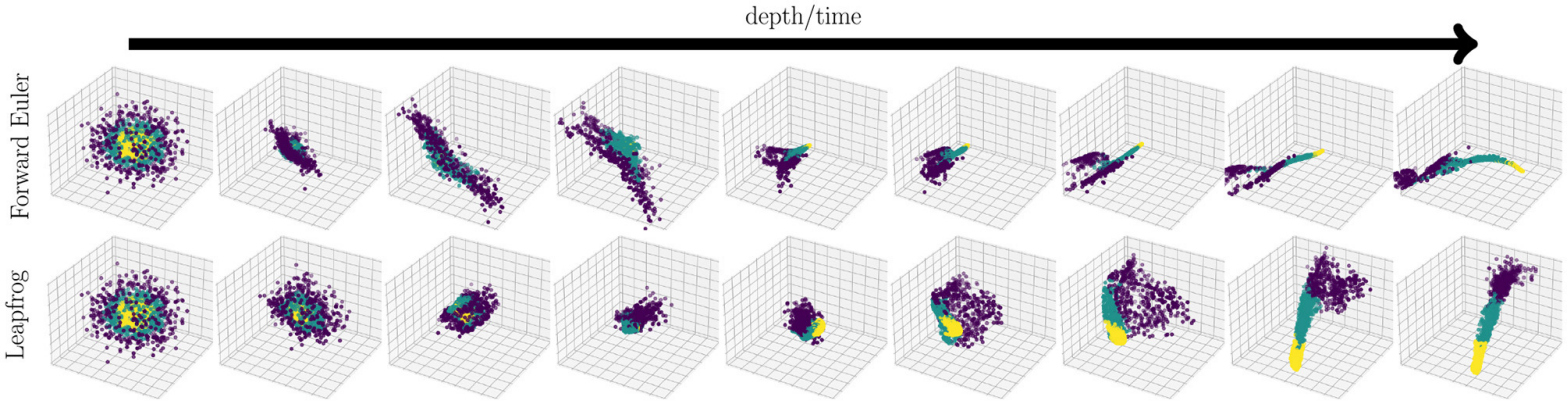
Comparison of feature trajectories for stable t-NNs with forward Euler (23b) and leapfrog integration (23a). Each image contains the features at a particularly layer of a trained network (layers *j* = 0, 4, …, 32). The forward Euler network resulted in a test accuracy of 90% and the leapfrog network resulted in a test accuracy of 93.50%. As expected, the leapfrog trajectory is smoother and contains rotational dynamics. The colors are linearly separable at the last layer, indicating good classification performance.

We create a network with *d* = 32 layers and use a step size of *h* = 2. We use the discrete cosine transform for **M**. We train for 50 epochs using Adam (Kingma and Ba, 2015) with a batch size of 10 and a learning rate of γ = 10^−2^. To create smoother dynamics, we regularize the weights using Equation (18) with regularization parameter λ = 10^−4^/*h*. We illustrate the dynamics of trained networks in Figure 4.

The dynamics in Figure 4 show the topological benefits of leapfrog integration. The data points exhibit smoother, rotational dynamics to reach the desired linearly-separable final configuration. In comparison, forward Euler propagation significantly changes topology during forward propagation. Such topological changes may yield ill-posed learning problems and poor network generalization.

## 6 Numerical results

We present two image classification problems to compare tensor linear layers and stable tensor neural networks to comparable matrix versions. Overall, the results show that t-NN trained with tubal loss functions generalize better to unseen data than equivalent matrix networks, and can do so with 20–30 times fewer network weights.

### 6.1 Experiment setup and hardware

We implement both tensor and matrix frameworks using PyTorch (RRID:SCR_018536) (Paszke et al., 2017). All of the code to reproduce the experiments is provided in https://github.com/elizabethnewman/tnn. All experiments were run on an Exxact server with four RTX A6000 GPUs, each with 48 GB of RAM. Only one GPU was used to generate each result. The results we report are for the networks that yielded the best accuracy on the validation data.

We train all models using the stochastic gradient method Adam (Kingma and Ba, 2015), which uses a default learning rate of 10^−3^. In most cases, we select hyperparameters and weight initialization based on the default settings in PyTorch. We indicate the few exceptions to this at the start of the corresponding sections. We also pair the stochastic optimizers with a learning rate scheduler that decreases the learning rate by a factor of γ every *M* steps. In all cases, we used the default γ = 0.9 and *M* = 100. This is a common practice to ensure convergence of stochastic optimizer in idealized settings (Bottou et al., 2018). We utilize a weight decay parameter in some experiments to reduce overfitting.

For the tensor networks in all experiments, we use the discrete cosine transform for **M**, which is a close, real-valued version of the discrete Fourier transform (DFT). The DFT matrix corresponds to the t-product, which has been shown to be effective for natural image applications (Kilmer and Martin, 2011; Hao et al., 2013; Newman et al., 2018).

### 6.2 MNIST dimensionality reduction

The MNIST dataset (LeCun et al., 2010) is composed of 28 × 28 grayscale images of handwritten digits. We train on 50, 000 images and reserve 10, 000 for validation. We report test accuracy on 10, 000 images not used for training nor validation. For NNs, we vectorize images and store as columns, resulting in a matrix of size 28^2^×*b* where *b* is the number of images. For t-NNs, we store the images as lateral slices, resulting in a tensor of size 28 × *b*×28.

**Figure 5:**
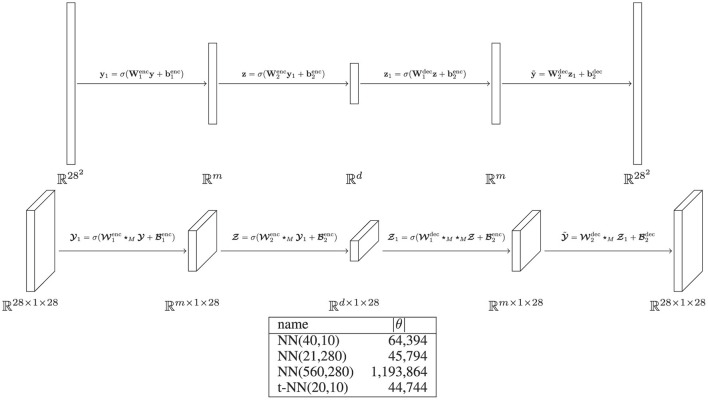
Description of MNIST autoencoder architectures with σ(*x*) = tanh(*x*). **(Top)** Four-layer matrix autoencoder NN(*m*,*d*) with first width *m* and latent space dimension *d*. **(Middle)** Four-layer tensor autoencoder t-NN(*m*,*d*) with first width *m* and latent space dimension *d*. For notational simplicity, we omit the vector lateral slice notation for the t-NN. **(Bottom)** Table of networks that we use. We pick sizes relative on the dimensions of t-NN(20,10). The first network NN(40,10) is given more features in the first layer of the encoder. The second network NN(21,280) is given the same number of latent space features and a corresponding width to have roughly the same number of weights as the t-NN. The third network NN(560,280) is given the same number of features on both layers as the t-NN.

**Figure 6:**
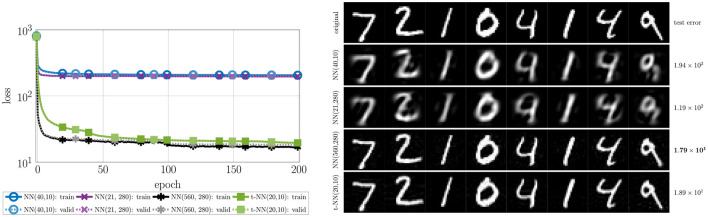
**(Left)** Convergence of the loss for the autoencoder example. The t-NN converges to a lower loss more quickly than the NNs. The validation loss closely follows the training loss, indicating good generalization. **(Right)** Autoencoder approximations to test images. The top row contains the true test images **y** and the subsequent rows contains the approximations to the true image $\tilde{\text{y}}=f_{\text{dec}}\left(f_{\text{end}}\left(\text{y},{\text{θ}}_{\text{enc}}\right),{\text{θ}}_{\text{dec}}\right)$ for various autoencoder architectures. To the right of each row, we report the average test error, $\frac{1}{\left|Y_{\text{test}}\right|}\underset{\text{y}\in Y_{\text{test}}}{\sum }||\text{y}-\tilde{\text{y}}|{|}_{2}$. Compared to the first two NN autoencoders, the t-NN produces clearer approximations and a test error an order of magnitude smaller. The autoencoder NN(560,280) does produce the smallest test error, but requires over 20 times more network weights than the t-NN autoencoder.

In this experiment, we train an autoencoder to efficiently represent the high-dimensional MNIST data in a low-dimensional subspace. Autoencoders can be thought of as nonlinear, parameterized extensions of the (truncated) singular value decomposition. Our goal is to solve the (unsrpervised) learning problem


(24) $$\begin{array}{l} \underset{{\text{θ}}_{\text{enc}},{\text{θ}}_{\text{dec}}}{\min}E_{\text{y}\sim Y}\;||f_{\text{dec}}\left(f_{\text{enc}}\left(\text{y},{\text{θ}}_{\text{enc}}\right),{\text{θ}}_{\text{dec}}\right)-\text{y}{||}_{2}^{2} \end{array}$$

where $f_{\text{enc}}:Y\to Z$ is the *encoder* and $f_{\text{dec}}:Z\to Y$ is the *decoder*. Here, Z is the latent space that is smaller than the data space; in terms of dimension, we say dim(Z)<dim(Y). Note that the mean squared error (MSE) tubal loss is the same as the MSE loss function when using an orthogonal transformation matrix, as we have done in our experiments. We describe the NN and t-NN autoencoder architectures used in Figure 5 and report results in Figure 6.

We observe that the t-NN outperforms the NN autoencoders with similar numbers of weights with an order of magnitude smaller training and validation loss and test error as well as qualitative improvements of the approximations. The neural network autoencoder with the same feature space dimensions, NN(560,280), performs best in terms of the loss and error metrics, but requires over 20 times more network weights than the t-NN autoencoder. The t-NN layers are able capture spatial correlations more effectively using multilinear operations, resulting in quality approximations with significantly fewer weights.

### 6.3 MNIST classification

We use the same MNIST dataset as for the autoencoder example. We train for 20 epochs using Adam with a batch size of 32 and a learning rate of 10^−2^. We add Tikhonov regularization (weight decay) with a regularization parameter of λ = 10^−4^. We use the PyTorch defaults for the other optimizer hyperparameters. For the t-cross entropy loss, we use a squared ℓ_2_-norm and normalize by the number of entries in the tube.

We compare four different two-layer neural network architectures, described in Table 1. We use either cross entropy loss or t-cross entropy loss, depending on the architecture.

**Table 1:** Description of MNIST two-layer network architectures with σ(*x*) = tanh(*x*).

**Name**	**Architecture**	**Layer 1**	**Layer 2**	**|θ|**
NN	**W**_2_σ(**W**_1_**y**_0_+**b**_1_)+**b**_2_	**W**_1_: 39 × 28^2^	**W**_2_: 10 × 39	31, 015
		**b**_1_: 39 × 1	**b**_2_: 10 × 1	
NN, square	**W**_2_σ(**W**_1_**y**_0_+**b**_1_)+**b**_2_	**W**_1_: 28^2^×28^2^	**W**_2_: 10 × 28^2^	623, 290
		**b**_1_: 28^2^×1	**b**_2_: 10 × 1	
t-NN	${\text{W}}_{2}\text{unfold}\left(\sigma \left(W_{1}⋆M{\vec{Y}}_{0}+{\vec{B}}_{1}\right)\right)+{\text{b}}_{2}$	$W_{1}$: 28 × 28 × 28	**W**_2_: 10 × 28^2^	30, 586
		${\vec{B}}_{1}$: 28 × 1 × 28	**b**_2_: 10 × 1	
t-NN, t-loss	$W_{2}⋆M\sigma \left(W_{1}⋆M{\vec{Y}}_{0}+{\vec{B}}_{1}\right)+{\vec{B}}_{2}$	$W_{1}$: 28 × 28 × 28	$W_{2}$: 10 × 28 × 28	30, 856
		${\vec{B}}_{1}$: 28 × 1 × 28	${\vec{B}}_{2}$: 10 × 1 × 28	

The t-NN architectures were chosen to preserve the size of the images. The NN width of 39 is chosen to be as small as possible such that the NN architecture does not have fewer parameters than the t-NN architecture. The final column reports the total number of learnable weights.

**Figure 7:**
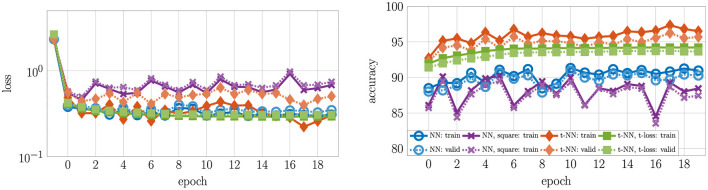
Loss and accuracy convergence for MNIST with four different two-layer neural networks. To see the differences clearly, we omit the initial accuracy, which was close to 10% for each network. The t-NN with cross entropy loss (orange diamonds 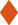) produces the best training (darker, solid) and validation (lighter, dashed) accuracy. The t-NN with t-loss (green squares 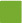) performs second best with in terms of accuracy, demonstrating the benefits of tensor operator layers. Despite having the greatest number of weights, the NN with square weights (purple × 's) performs worst in terms of accuracy.

We report the convergence and accuracy results in Figure 7 and Table 2, respectively.

**Table 2:** MNIST training, validation, and test accuracy per class and overall for the four architectures.

		**0**	**1**	**2**	**3**	**4**	**5**	**6**	**7**	**8**	**9**	**Overall**
Train	NN	97.05	96.54	90.58	89.73	93.03	88.91	92.36	91.91	87.79	84.57	91.33
		NN, square	96.67	93.85	92.58	84.82	94.84	89.11	97.91	88.76	77.98	85.56	90.24
		t-NN	**98.98**	**99.64**	**97.12**	**97.88**	**98.29**	**96.06**	**98.68**	**97.60**	**93.30**	**95.59**	**97.36**
		t-NN, t-loss	97.28	97.06	95.97	91.09	94.92	92.50	97.26	92.26	91.99	91.61	94.22
Valid	NN	96.90	96.46	89.50	89.79	92.13	89.94	92.28	91.33	88.85	81.02	90.94
		NN, square	94.50	93.36	90.60	84.68	94.99	89.83	**97.36**	89.26	77.81	82.16	89.52
		t-NN	**97.60**	**99.29**	**95.40**	**97.01**	**97.34**	**95.66**	96.75	**97.08**	**92.47**	**93.26**	**96.26**
		t-NN, t-loss	96.20	97.08	94.80	90.94	93.46	93.60	97.26	92.46	92.16	89.21	93.76
Test	NN	98.16	97.18	89.44	92.18	93.08	88.00	90.50	89.49	87.58	85.43	91.20
		NN, square	97.14	94.89	92.44	85.84	95.01	88.79	**97.49**	87.45	78.23	83.35	90.11
		t-NN	**98.67**	**99.38**	**95.25**	**97.03**	**98.37**	**94.96**	97.08	**96.50**	93.74	**94.05**	**96.55**
		t-NN, t-loss	98.16	98.33	95.35	93.66	95.21	91.59	97.18	90.66	**93.94**	91.08	94.57

The t-NN architecture with cross entropy loss consistently produces the highest accuracy. The bolded values indicate the highest accuracy for the class and dataset.

**Figure 8:**
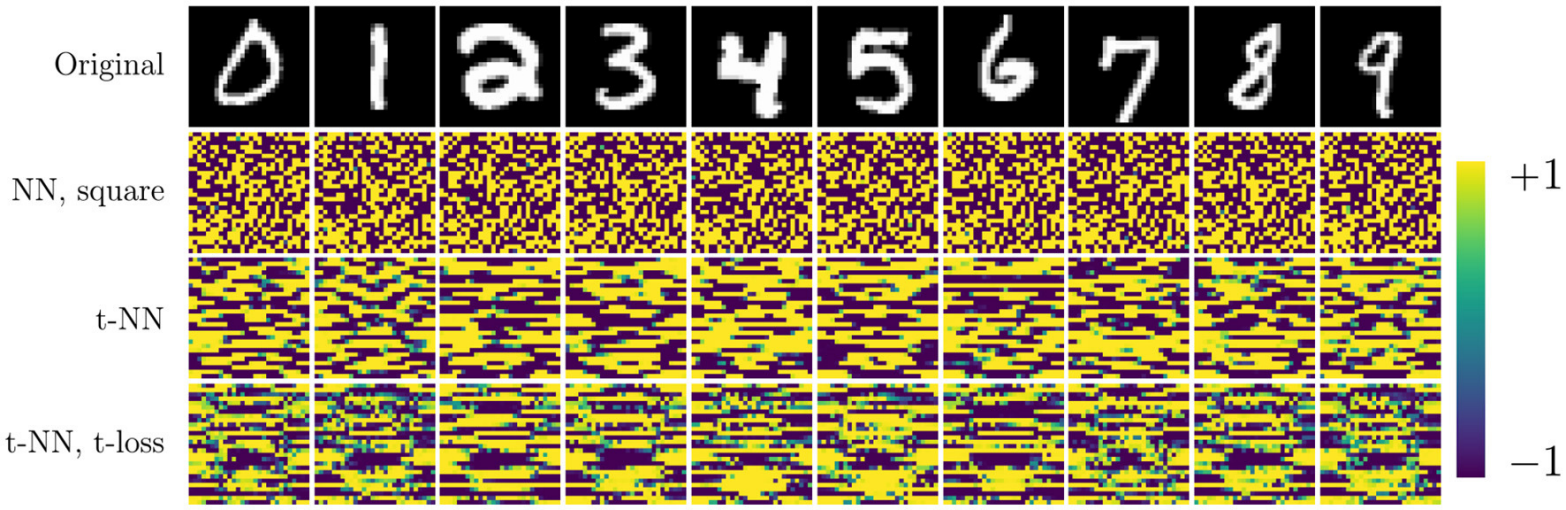
Features from first layer of NN, square, t-NN with cross entropy, and t-NN with t-cross entropy networks. Both t-NN features contain more structure because the ⋆_*M*_-operation respects spatial correlations.

The t-NN architecture with cross entropy loss outperforms all networks in terms of test accuracy and accuracy per class. The second-best performing network is the t-NN with t-loss. These results are evidence that the features learned from the tensor linear layer (layer 1) are better than those learned by a dense matrix layer. We further note that matrix network NN with square weights has the same final layer shape as the t-NN with cross entropy loss; the only difference between the networks is the first layer. We depict the learned features of each network that preserves the size of the images in Figure 8. The t-NN features from the first layer contain more structure than the NN features with square weights. This reflects the ⋆_*M*_-operation, which first acts along the tubes (rows of the images in Figure 8). We also observe that the features of NN are more extreme, close to +1 and −1, the limits of the range of the activation function σ(*x*) = tanh(*x*). In comparison, the features extracted from the t-NN with t-loss offer more variety of entries, but still hits the extreme values often. This demonstrates that t-NNs still produce rich feature spaces and are able to achieve these features with about 20 times fewer weights.

**Figure 9:**
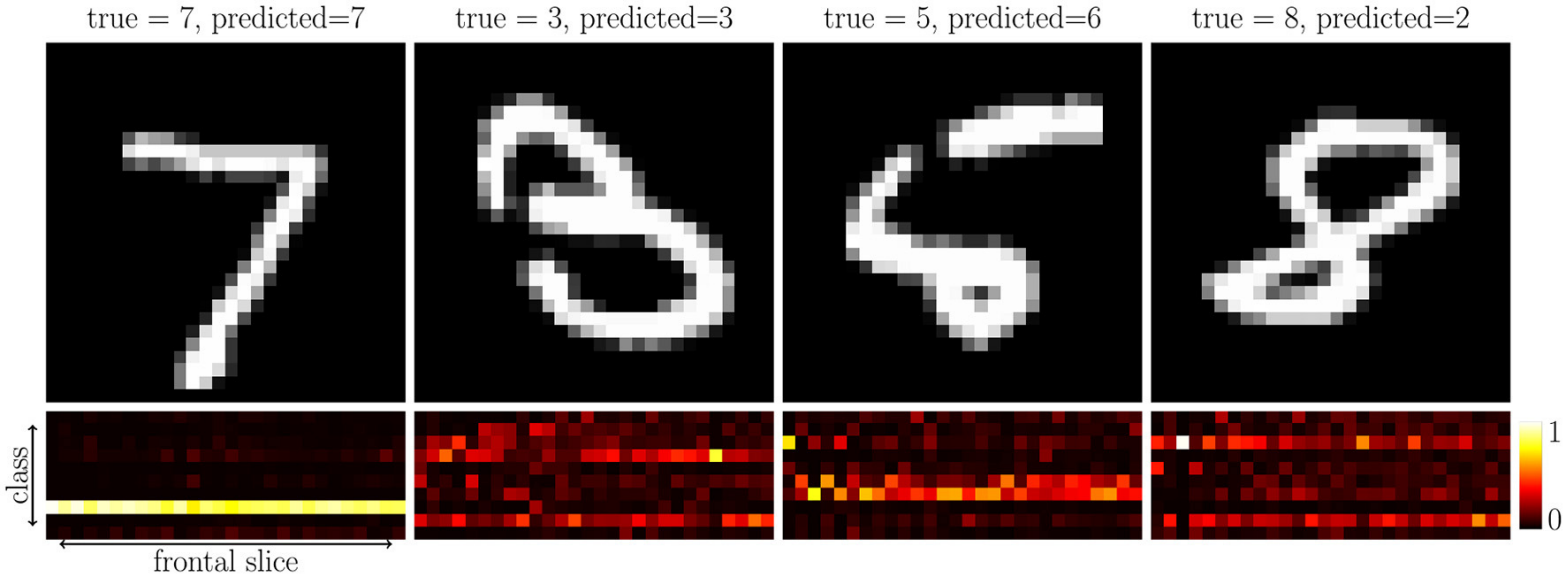
Illustration of t-softmax of MNIST test images using t-NN with t-loss. The top row are the test images $\vec{Y}\in {\mathbb{R}}^{28\times 1\times 28}$ and the bottom row are the values of the tubal softmax of the output $F_{\text{tnn}}\left(\vec{Y},\text{θ}\right)\in {\mathbb{R}}^{10\times 1\times 28}$, shown in the transform domain. Each row of the tubal softmax images corresponds to a different class and each column to a different frontal slice. The row with the largest ℓ_2_-norm corresponds to the predicted class, where the top row corresponds to class 0 and the bottom row corresponds to class 9. The left two images were predicted correctly and the right two images were predicted incorrectly.

We note that the t-NN network with t-cross entropy loss performs well, and we gain insight into the beneifts of t-losses from the accuracy per class in Figure 9. We observe that when we use tubal losses, we require high values for many frontal slices. This creates a more rigorous classification requirement and, as we will see in subsequent experiments, can yield networks that generalize better. Additionally, the distribution of values in the tubal softmax function is reflective of the predicted class. For the second image (true = 3), the two most likely predicted classes were, in order, 3 and 8. Qualitatively, the particularly handwritten 3 has similarities to the digit 8, and the tubal softmax captures this similarity. For the cases that were incorrectly predicted the digit, the handwritten image had structure emblematic of the predicted class and second most likely predicted class matched the true label.

### 6.4 CIFAR-10

The CIFAR10 dataset (Krizhevsky and Hinton, 2009) is composed of 32 × 32 × 3 RGB natural images belonging to ten classes. We train on 40, 000 images and reserve 10, 000 for validation. We report test accuracy on 10, 000 images not used for training nor validation. For NNs, we vectorize images and store as columns, resulting in a matrix of size (3·32^2^) × *b* where *b* is the number of images. For t-NNs, we store the images as lateral slices and stack the color channels vertically, resulting in a tensor of size (3·32) × *b*×32. We train for 500 epochs using Adam with a batch size of 32. We use a learning rate of 10^−3^ that decays after every 100 epochs by a factor of 0.9. We use the PyTorch defaults for the other optimizer hyperparameters. For the t-cross entropy loss, we use a squared ℓ_2_-norm and normalize by the number of entries in the tube.

**Figure 10:**
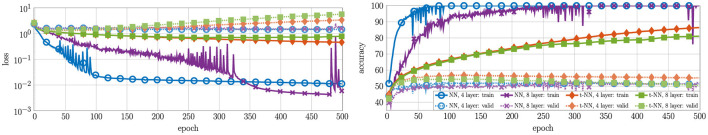
Loss and accuracy convergence for CIFAR-10 with different Hamiltonian network depths. We only show the convergence for the t-NN with t-cross entropy loss, which achieved a top validation accuracy of at least 54.37%, compared to the t-NN with cross entropy loss, which topped out at 54.32%. For the accuracy, we start with epoch 5 to highlight the differences between networks.

We compare the performance of the Hamiltonian networks with dense matrix operators and dense tensor operators for various numbers of layers (*d* = 4, 8). In conjunction with the Hamiltonian network, we use the smoothing regularizer (20) with a regularization parameter of λ = 10^−2^. We describe the network architectures and number of parameters in Table 3. The NN architectures require more than 30 times the number of weights than the t-NN architectures. We compare the convergence and accuracy results in Figure 10 and Table 4, respectively.

**Table 3:** Description of CIFAR-10 Hamiltonian network architectures with σ(*x*) = tanh(*x*).

**Name**	**Hamiltonian layers**	**Final layer**	**|θ|**
NN	**W**_*j*_: (3·32^2^) × (3·32^2^)	**W**_*d*+1_: 10 × 3072	4 layers: 37, 779, 470
	***b*_*j*_: 1 × 1**	**b_*d*+1_: 10 × 1**	**8 layers: 75, 528, 210**
t-NN	**W**_*j*_: (3·32) × (3·32) × 32	**W**_*d*+1_: 10 × (3·32^2^)	4 layers: 1, 210, 506
	**b**_*j*_: 1 × 1 × 32	**b**_*d*+1_: 10 × 1	8 layers: 2, 390, 282
t-NN, t-loss	$W_{j}$: (3·32) × (3·32) × 32	$W_{d+1}$: 10 × (3·32) × 32	4 layers: 1, 210, 816
	${\vec{B}}_{j}$: 1 × 1 × 32	${\vec{B}}_{d+1}$: 10 × 1 × 32	8 layers: 2, 390, 592

The architectures were chosen to preserve the sizes of the CIFAR-10 images. The final column |***θ***| reports the total number of learnable weights.

**Table 4:** CIFAR-10 training, validation, and test accuracy per class and overall for the four architectures.

		**Plane**	**Car**	**Bird**	**Cat**	**Deer**	**Dog**	**Frog**	**Horse**	**Ship**	**Truck**	**Overall**
Train	NN4	**99.95**	**100.00**	**99.95**	**100.00**	**99.98**	**99.97**	**100.00**	**99.97**	**100.00**	**100.00**	**99.98**
		NN8	99.90	99.97	99.80	99.95	99.93	100.00	100.00	100.00	99.93	99.93	99.94
		t-NN4	74.12	79.97	60.44	43.02	59.82	65.27	65.66	88.47	77.14	74.83	68.84
		t-NN8	76.09	81.15	61.12	35.80	69.04	58.83	62.52	91.45	76.94	75.28	68.79
Valid	NN4	54.57	62.77	40.85	29.58	39.36	42.77	58.24	59.52	**70.84**	60.06	51.99
		NN8	**62.08**	65.54	39.76	**33.92**	38.53	43.36	**62.34**	56.62	70.74	59.06	53.30
		t-NN4	59.51	68.32	**49.11**	32.68	49.53	**52.25**	51.41	**73.62**	67.64	**62.86**	**56.83**
		t-NN8	60.43	**66.63**	47.12	23.37	**53.79**	44.82	47.90	73.24	65.33	59.86	54.37
Test	NN4	57.30	64.70	40.60	28.10	38.30	42.10	57.30	58.20	**69.20**	57.90	51.37
		NN8	**62.60**	63.10	40.70	33.20	39.10	44.80	**61.60**	55.30	69.00	55.80	52.52
		t-NN4	61.00	66.90	48.40	**34.40**	48.10	**54.70**	54.50	72.50	67.30	**63.30**	**57.11**
		t-NN8	**62.60**	**67.30**	**48.70**	25.20	**52.50**	46.70	50.20	**72.90**	63.40	60.10	54.96

The t-NN architectures with t-cross entropy loss produce the highest overall validation and test accuracy, the traditional metrics to indicate generalization ability. The bolded values indicate the highest accuracy for the class and dataset.

There are several key takeaways from the numerical results. First, the depth of the network did not significantly change performance in this experiment. We state this observation cautiously. We observe this behavior for a certain set of fixed hyperparameters (e.g., step size *h*, learning rate, regularization parameter λ,...). The interaction of the hyperparameters and performance is complex and a complete ablation study is outside of the scope of this paper. A second takeaway is that the t-NN trained with the tubal loss generalizes better the NN networks and better than t-NNs with cross entropy loss (not shown for simplicity; see Figure 10 for details). This behavior is especially apparent when looking at the test loss in Table 4. The t-NN with four Hamiltonian layers and t-loss performs well overall, obtaining almost 5% better overall test accuracy. In comparison, the matrix NN quickly overfits the training data and thus does not generalize as well. In terms of the test accuracy per class, the t-NN architectures achieve the best performance in all but two classes.

**Figure 11:**
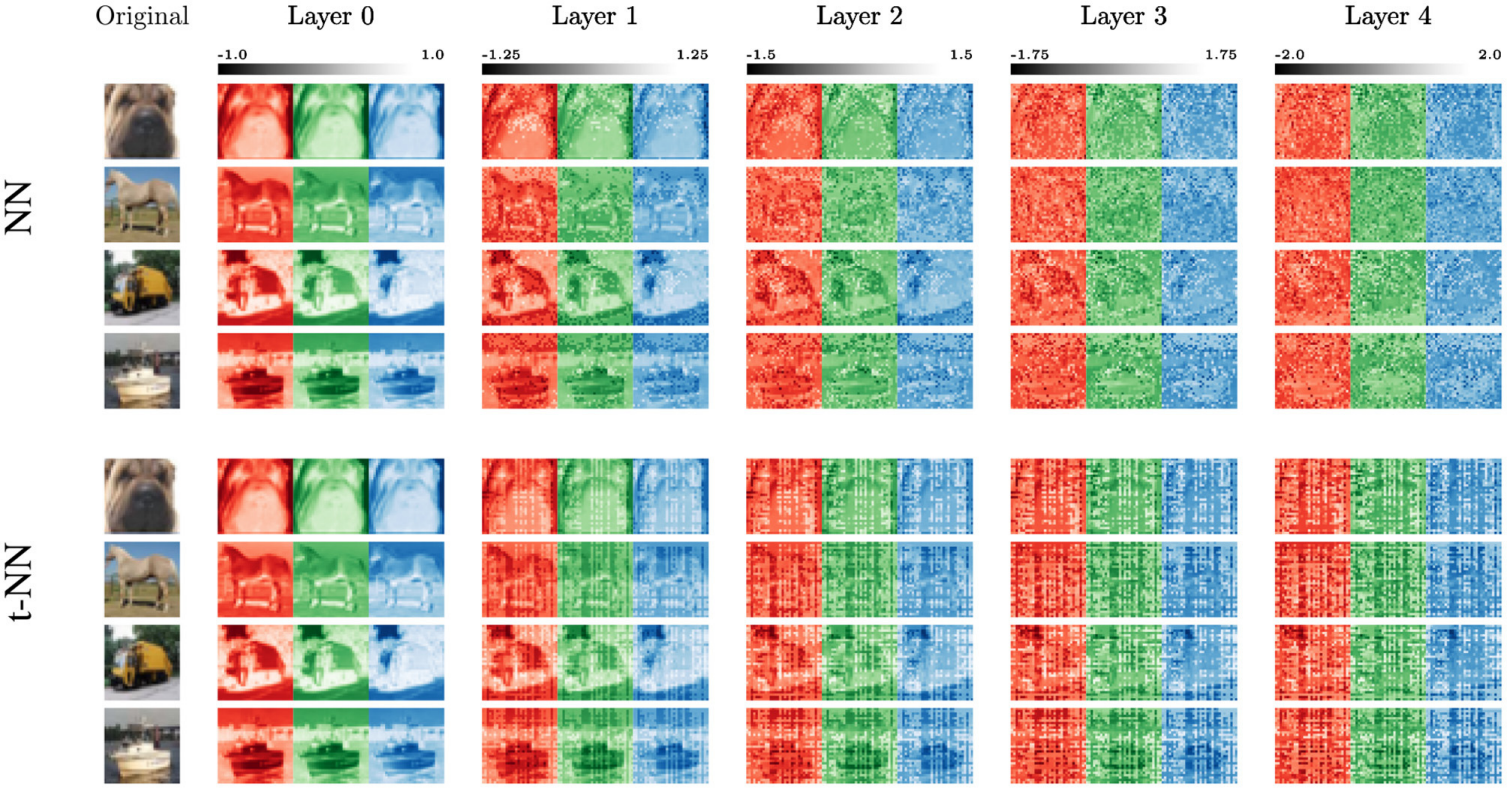
Features from the trained 4-layered Hamiltonian networks of both the matrix and tensor parameterized cases for four different training images (top-to-bottom: dog, horse, truck, ship). For the t-NN, we use the better-performing network with t-cross entropy loss. Here, Layer 0 shows the separated color channels of the original images.

To look into the performance further, we examine the extracted features of Hamiltonian NNs and t-NNs in Figure 11. We see that the NN and t-NN features share similarities. Both features gradually remove the structure of the original image at similar rates. The t-NN architecture achieves this pattern with significantly fewer network weights (over 30 times fewer). The noisy artifacts differ between the two architectures. In particular, we see that the t-NN layers produce blockier artifacts because of the structured ⋆_*M*_-operation.

**Figure 12:**
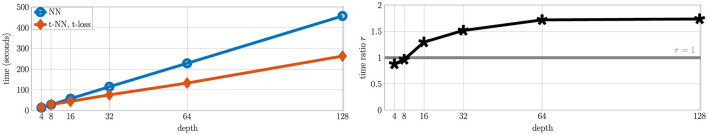
**(Left)** Average time per epoch to train a Hamiltonian network for a fixed batch size. We ran each depth for five epochs and the maximum standard deviation of time was on the order of 10^−2^ relative to the average time. **(Right)** Time ratio average *r* = NN epoch/t-NN epoch. As the depth of the network grows, the time per epoch for NNs takes almost 1.75 times longer than for t-NNs.

In the last numerical study in Figure 12, we explore how quickly we can train Hamiltonian t-NNs compared to NNs. In addition to an order of magnitude fewer weights, training t-NNs takes less time than training NNs. As we increase the depth of the networks, we see that each NN epoch takes approximately 1.75 times longer to complete. This performance could potentially be further improved if we optimized the ⋆_*M*_-product, e.g., using fast transforms instead of matrix multiplication.

### 6.5 CIFAR-100

**Figure 13:**
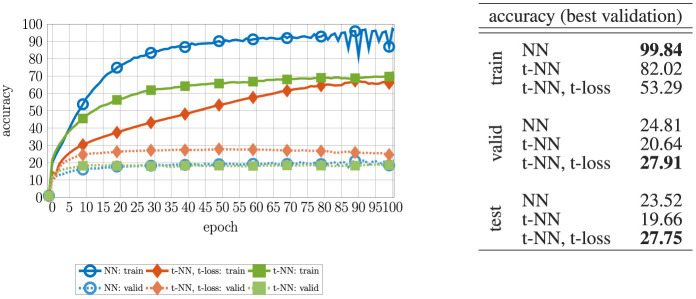
**(Left)** Convergence of the accuracy for the CIFAR-100 experiment. To delineate the performance on the validation data, we show the first 100 epochs out of 500 total. The t-NN with t-loss converges to the highest validation accuracy and avoids the generalization gap longest out of all presented networks. **(Right)** Final accuracy for the training, validation, and test data. We report the results using the networks that produced the highest validation accuracy during training.

The CIFAR100 dataset (Krizhevsky and Hinton, 2009) is composed of 32 × 32 × 3 RGB natural images belonging to 100 classes. We train on 40, 000 images and reserve 10, 000 for validation. We report test accuracy on 10, 000 images not used for training nor validation. We use the same setup and training parameters as the CIFAR10 experiment (Section 6.4). For all experiments, we use Hamiltonian networks with a depth of *d* = 16, a step size of *h* = 0.25, and a regularization parameter λ = 10^−2^. We report the accuracy results in Figure 13.

Observing the results in Figure 13, the conclusions are the same as in the CIFAR-10 experiment. Specifically, training with the t-NN and t-loss produces the best test accuracy, about a 4% improvement from the comparable NN network. This demonstrates that the benefits of dense tensor operations over dense matrix operations can be realized for more challenging classification problems and motivates further development of these tools to improve state-of-the-art convolutional neural networks and other architectures.

## 7 Conclusion

We presented tensor neural networks (t-NNs) as a new approach to parameterize fully-connected neural networks. We operate using the ⋆_*M*_-product which can reduce the number of network weights by an order of magnitude while maintaining the same expressiveness. We introduced tubal loss functions that are an algebraically-consistent t-NN architecture. Because the ⋆_*M*_-framework gives rise to a tensor algebra that preserves matrix properties, we extended the notion of stable neural networks to t-NNs, which enable the development of deeper, more expressive networks. Through numerical experiments on benchmark image classification tasks, we demonstrated that t-NNs offer a more efficient parameterization and, when trained with tubal loss functions, can generalize better to unseen data.

Our work opens the door to several natural extensions. First, we note that while this paper focused on imaging benchmark problems in machine learning, the ⋆_*M*_-framework can be applied to many data sets, including dynamic graphs (Malik et al., 2021), longitudinal omics data (Mor et al., 2022), and functional magnetic resonance imaging (fMRI) (Keegan et al., 2022). Second, we could use tensor parameterizations to improve convolutional neural networks (CNNs), just as we used t-NN layers to improve fully-connected networks. CNNs are state-of-the-art for image classification and rely on convolution operations. The ⋆_*M*_-product is, in some sense, a convolution based on the transformation **M**; in fact, when **M** is the discrete Fourier transform, the result is a circulant convolution. A t-CNN could offer more efficient parameterization and a greater range of convolutional features that could increase the expressibility of the network. Third, we could extend the use of tubal loss functions to any network architecture. Tubal loss functions offer more stringent requirements to fitting data which can mitigate overfitting. Additionally, tubal loss functions foster a new level of flexibility to evaluate performance, such as various norms to transform tubal probabilities into scalars and new measures of accuracy per frontal slice. Fourth, we can consider learning the operator **M** based on the data or allowing the operator to evolve with the layers. Lastly, we can explore methods to improve t-NN efficiency on CPUs and GPUs by exploiting the parallelize of the ⋆_*M*_-products.

## Data availability statement

The datasets presented in this study can be found in online repositories. The names of the repository/repositories and accession number(s) can be found at: https://pytorch.org/vision/main/datasets.html and https://github.com/elizabethnewman/tnn.

## Author contributions

EN: Conceptualization, Data curation, Formal analysis, Funding acquisition, Investigation, Methodology, Project administration, Resources, Software, Supervision, Validation, Visualization, Writing—original draft, Writing—review & editing. LH: Conceptualization, Data curation, Funding acquisition, Writing—original draft, Writing—review & editing. HA: Conceptualization, Data curation, Writing—original draft, Writing—review & editing. MK: Funding acquisition, Writing—original draft, Writing—review & editing.
